# Exploring Absenteeism in Medical Education: Perceptions of Students and Professors at the University of Granada

**DOI:** 10.1007/s40670-025-02482-w

**Published:** 2025-09-20

**Authors:** Maria Isabel Rodriguez-Lara, Carolina Torres, Francisco J. Amaro-Gahete, Franscisco Hernandez-Torres, Miguel A. Montero-Alonso, Maria Isabel Núñez, Aliaga Luis

**Affiliations:** 1 Department of Biochemistry and Molecular Biology III and Immunology, Faculty of Medicine, University of Granada, Granada, Spain; 2 Department of Physiology, Faculty of Medicine, Sport and Health University Research Institute (iMUDS), University of Granada, Granada, Spain; 3 Department of Statistics and Operations Research, Faculty of Medicine, University of Granada, Granada, Spain; 4 Department of Radiology and Physical Medicine, Faculty of Medicine, University of Granada, Granada, Spain; 5 Department of Medicine, Faculty of Medicine, University of Granada, Granada, Spain; 6 Instituto de Investigacion Biosanitaria, Ibs.Granada, Granada, Spain; 7 Centro de Investigacion Biomedica en Red Fisiopatologia de La Obesidad y Nutricion (CIBERobn), Instituto de Salud Carlos III, 28029 Madrid, Spain; 8 Biopathology and Regenerative Medicine Institute (IBIMER), Centre for Biomedical Research, University of Granada, Granada, Spain

**Keywords:** Student absenteeism, Medical education, Lecture attendance, Teaching methodologies, Student engagement

## Abstract

Student absenteeism in higher education has become a growing concern due to its potential impact on academic performance and professional development. This study aims to assess the prevalence of absenteeism in the Faculty of Medicine at the University of Granada (Spain) and to explore the perceptions of both students and professors regarding this issue. Attendance was recorded at two different points during the semester, and surveys were conducted among faculty members and students to gather qualitative and quantitative data. Results showed a significant decrease in attendance over the semester. Faculty members identified lack of student engagement and large class sizes as key issues, while students highlighted poor teaching methodologies and excessive workload as the main reasons for non-attendance. The findings suggest that improving teaching methods, increasing student engagement, and restructuring class formats may help to reduce absenteeism.

**Supplementary Information:**

The online version contains supplementary material available at 10.1007/s40670-025-02482-w.

## Introduction

Student absenteeism is a widespread issue across universities and affects numerous faculties, posing challenges to academic performance and student engagement. Despite strict attendance policies, absenteeism remains a significant problem in medical and health sciences education, involving undergraduate students worldwide [1, 2]. This is of particular concern in medical schools, where attendance is often considered essential for the acquisition of both basic and clinical knowledge [3, 4].

To better understand the underlying causes of absenteeism, this study draws upon established educational theories. Self-Determination Theory (SDT), a macrotheory of human motivation, posits that student engagement is closely tied to the satisfaction of three basic psychological needs: autonomy, competence, and relatedness. When these needs are supported by the learning environment—through teaching styles that promote autonomy, meaningful interaction, and constructive feedback, students are more likely to exhibit intrinsic motivation and active participation. Conversely, environments perceived as controlling or disengaging may lead to disaffection, passivity, or even resistance, which can manifest as absenteeism. In this context, absenteeism may be interpreted not merely as a behavioral issue, but as a visible indicator of a deeper motivational disconnect. This perspective aligns with Student Engagement Theory, which emphasizes the importance of emotional and cognitive investment in learning activities as a predictor of academic success [5, 6].

Several studies have also linked absenteeism to poorer academic and clinical outcomes. For instance, missing key sessions has been associated with lower academic performance, reduced clinical competencies such as prescription writing, and even concerns about professionalism [7–11].

These findings underscore the broader implications of disengagement in medical education, beyond theoretical knowledge acquisition.

While absenteeism in medical education has been widely studied in international contexts—particularly in countries such as the USA, the UK, and various Middle Eastern and Asian nations—these studies often focus on either student or faculty perspectives and are typically framed within institutional policies or cultural norms specific to those regions. In contrast, the Spanish academic context has received comparatively little attention in the literature, with only a handful of studies addressing absenteeism in undergraduate medical programs. Moreover, few of these adopt a dual-perspective approach that integrates both student and faculty viewpoints. This study aims to fill that gap by offering a comprehensive, context-specific analysis of absenteeism at a Spanish medical school, contributing to the broader international discourse while addressing a local need for evidence-based educational strategies.

Understanding the underlying causes of absenteeism is crucial for developing effective strategies to enhance student engagement and academic success. Factors influencing attendance include the perceived effectiveness of lectures, the availability of recorded sessions and online resources, scheduling conflicts, personal motivation, and institutional policies. Addressing these elements requires a nuanced understanding of both student and faculty perspectives within the specific educational context.

## Methods

### Study Design

An observational study was conducted at the Faculty of Medicine of the University of Granada. Class attendance was recorded on two strategically selected dates within the semester: March 7 and May 7 in the academic years 2023–2024. These assessments were carried out without prior notice with the objective of observing any potential variations in attendance as the course progressed. The assessments were carried out at a time considered to be the highest attendance slot in all courses (10:00–11:30).

To assess the point of view, surveys were distributed to both faculty members (*n* = 278) and students (*n* = 1360) to collect data on their perceptions of absenteeism (Supplementary material). The survey was conducted using LimeSurvey software, an internally hosted, free, and open-source online survey application at the University of Granada. LimeSurvey surpasses other survey tools due to its extensive set of question types, advanced branching features, and a robust survey management system with comprehensive statistical analysis capabilities.

The instruments were designed by the research team based on a review of relevant literature and were internally reviewed to ensure clarity and alignment with the study objectives. Although formal psychometric validation was not conducted, the questionnaires were piloted with a group of student representatives (class delegates), who provided feedback on item clarity and contextual relevance. The questionnaire was designed to include dichotomous, multiple choice, and open-ended questions. The teacher’s survey was sent through the Dean’s Office to the entire faculty. To facilitate student access to the survey, a QR code was provided at the time of attendance registration. Additionally, the survey link was shared through the Student Commission to capture the responses from those not present in the classroom.

The survey for faculty members included questions about their views on absenteeism, potential solutions, and their teaching methods. The student survey focused on reasons for non-attendance, preferred learning methods, and opinions on lecture effectiveness. Quantitative data were analyzed using descriptive statistics. Regarding the qualitative analysis, two independent researchers conducted an inductive thematic analysis of the open-ended responses. Discrepancies were resolved through discussion until consensus was reached. While inter-coder reliability was not formally calculated, agreement between coders was consistently high.

In this study, the dependent variable was absenteeism while age, gender, and year of study were the independent variables. Participation in the survey was entirely voluntary, and respondents were informed that their answers would be used for research purposes. The anonymity of the participants was strictly maintained, and no identifiable information was collected. The study adhered to ethical guidelines for research involving human subjects, ensuring that participants’ rights and privacy were protected throughout the research process.

### Statistical Analysis

Continuous variables are presented as median and interquartile range. Categorical variables are reported as proportions and their 95% confidence interval. The chi-square test is used to compare categorical variables. *p* values ≤ 0.05 are taken as significant. Statistical calculations are made with SPSS version 28.0.1.

## Results

### Faculty Members

A total of 107 professors participated in the survey, 38.5% of the 278 medical school professors. Of these, 65 were male (69.9%) and 25 female (26.9%), with a median age of 60 years (interquartile range: 47–64). Regarding teaching experience, 46 faculty members (43.0%) had ≥ 20 years of teaching experience. In terms of teaching load, 41 faculty members taught one subject (44.09%), 38 taught two (40.86%), 11 taught three (11.83%), and 3 taught four or more subjects (3.23%). The distribution of faculty by course is shown in Table 1.

**Table 1:** Distribution of faculty members by course. The 95% CI (confidence interval) for a proportion was calculated using theAgresti-Coull method. The percentages are based on a total of 107 faculty responses, and the sum exceeds 100% as some professors teach in more than one course

Course	Faculty members, *n* (%)	95% CI
First	22 (20.6%)	(14.0%, 29.3%)
Second	35 (32.7%)	(24.6%, 42.1%)
Third	24 (22.4%)	(15.6%, 31.3%)
Fourth	28 (26.2%)	(18.7%, 35.3%)
Fifth	27 (25.2%)	(17.8%, 34.4%)

**Fig. 1:**
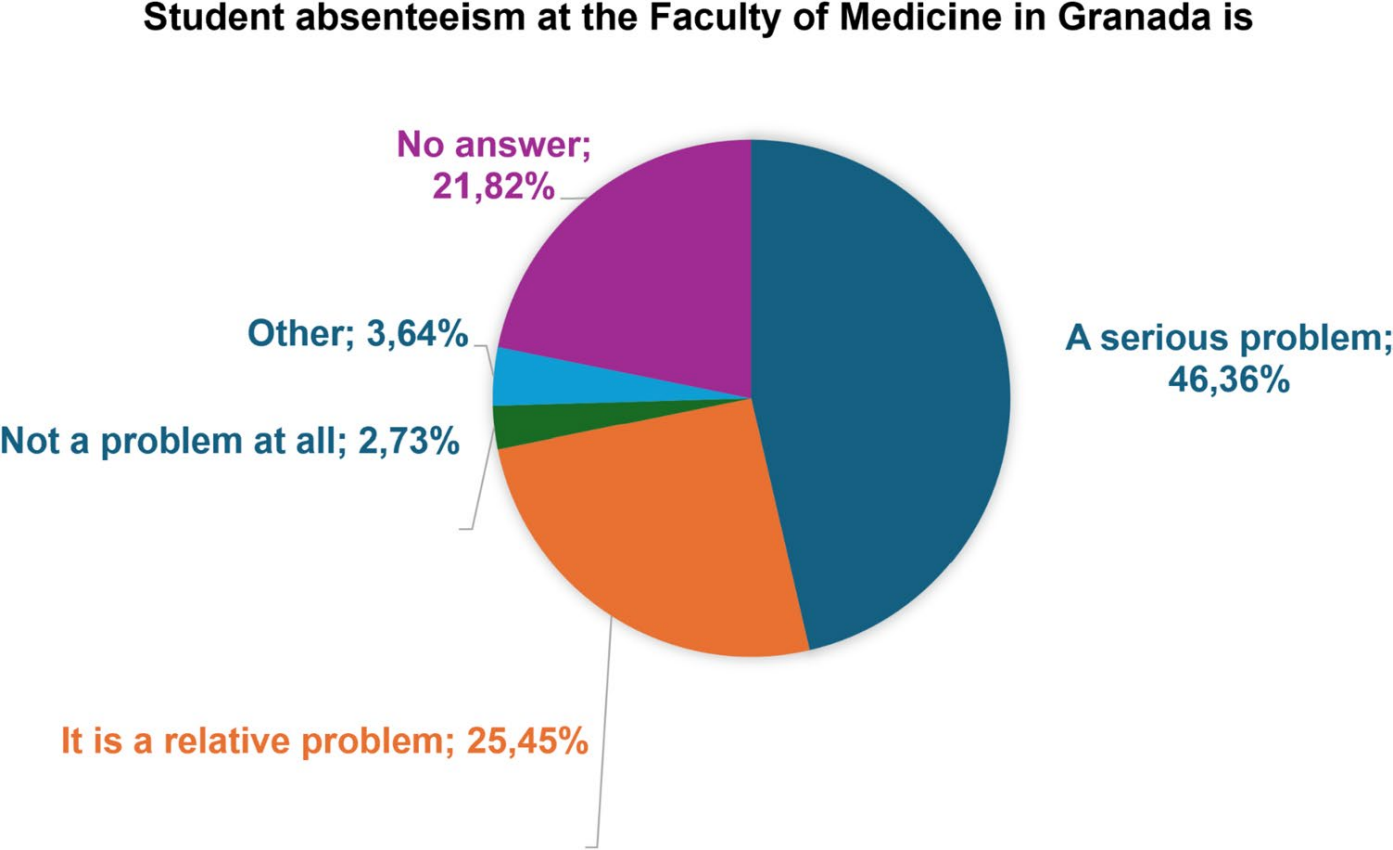
How professors feel about missing classes

Fifty teachers (46.36%) considered student absenteeism to be a serious problem, and 27 (25.45%) considered it to be a relative problem (Fig. 1).

**Fig. 2:**
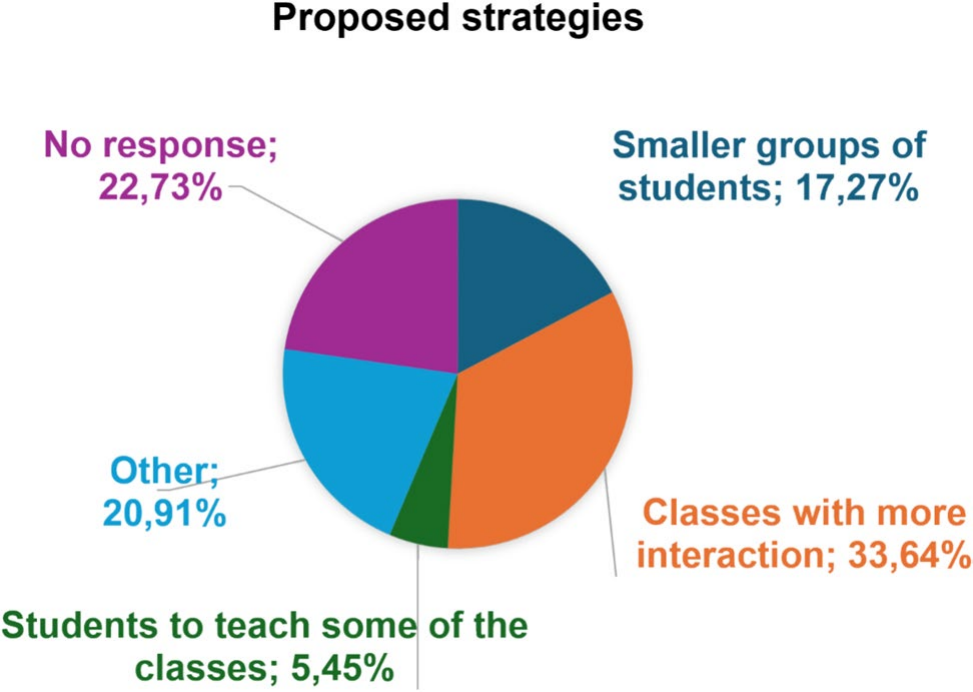
Measures proposed by teachers to address absenteeism problems

Furthermore, when the professors were asked which solutions they considered most important to reduce student absenteeism in this faculty, classes with more teacher-student interaction (33.64%) and smaller groups of students (17.27%) were the most highly valued solutions (Fig. 2).

The analysis of free-text responses from professors regarding student absenteeism in classes revealed several common themes and notable suggestions: professors suggested changing the curriculum to focus less on evaluation and exam preparation (e.g., MIR) and more on practical teaching and seminars. Increasing interaction between professors and students was also seen as crucial. This includes revising course content, providing academic guidance, and making classes more engaging. Reducing class sizes was also a recurring theme. Smaller groups are believed to facilitate better interaction and personalized instruction. Some professors reported that taking attendance and valuing class participation could incentivize students to attend. However, others consider this not to be an adequate solution. The lack of student motivation appeared also as a recurring theme. It was suggested that classes should be designed to be more relevant and practical to capture students’ interest. The need for innovation in teaching techniques was also highlighted. This includes adopting new methodologies, using technology, and making classes more interactive.

Professors emphasized the importance of instilling a sense of responsibility and accountability in students. This includes evaluating students’ participation and encouraging their active involvement in learning. Improving the quality of teaching through faculty development programs was also suggested. This includes training in modern pedagogical techniques and continuous professional development.

#### Students

The total number of students enrolled in the courses where attendance was recorded was 1360. Of these, 692 (50.9%) were present in class on the assessment day, with their distribution by academic year shown in Table 2.

**Table 2:** Total enrolled students by course and attendance on the first day of attendance registration (March 7,2024). Values are presented as number of students attending (*n*) and the corresponding percentage (%) of enrolled students for that academic year. The 95% CI (confidence interval) for a proportion was calculated using theAgresti-Coull method

Academic year	Enrolled students	Attendance, *n* (%)	95% CI
First	313	218 (69.7%)	(64.3%, 74.5%)
Second	280	117 (41.8%)	(36.1%, 47.7%)
Third	314	119 (37.9%)	(32.7%, 43.4%)
Fourth	285	185 (64.9%)	(59.2%, 70.2%)
Fifth	168	53 (31.5%)	(25.1%, 39.0%)
Total	1360	692 (50.9%)	(48.2%, 53.5%)

In the second assessment of attendance, the number of enrolled students was 1401. Attendance dropped from 50.9% in March to 36.7% in May (Table 3). The differences in class attendance between the first and second assessments were statistically significant (*p* < 0.001).

**Table 3:** Total enrolled students by course and attendance on the second day of attendance registration. (May 7, 2024).Values are presented as number of students attending (*n*) and the corresponding percentage (%) of enrolled students for that academic year. The 95% CI (confidence interval) for a proportion was calculated using the Agresti-Coull method

Academic year	Enrolled students	Attendance, *n* (%)	95% CI
First	313	83 (26.5%)	(21.9%, 31.7%)
Second	321	117 (36.4%)	(31.4%, 41.9%)
Third	314	97 (30.9%)	(26.0%, 36.2%)
Fourth	285	152 (53.3%)	(47.5%, 59.0%)
Fifth	168	65 (38.7%)	(31.7%, 46.2%)
Total	1401	514 (36.7%)	(34.2%, 39.2%)

A total of 789 students responded to the survey in the first attendance assessment, with a median age of 20 years (interquartile range: 19–22). Among them, 235 (29.9%) were male, and 483 (61.2%) were female. One hundred and 44 students (18.0%) did not respond to any of these questions, likely due to technical issues. Regarding year of enrollment, most students were in their first year (240/789), followed by the fourth year (184/789).

**Fig. 3:**
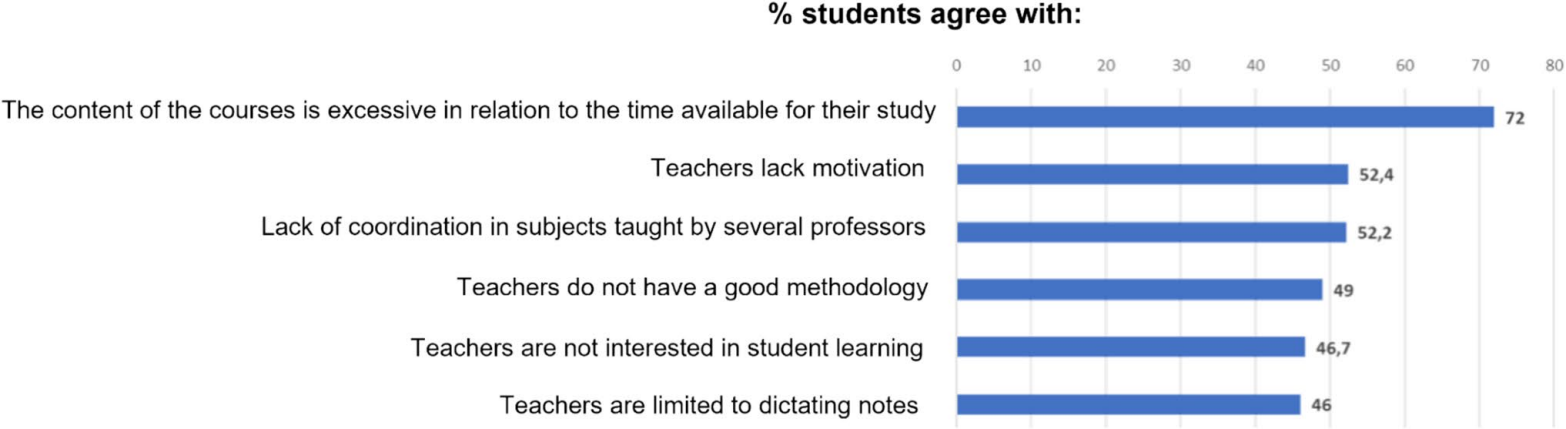
Reasons given by students on the aspects that cause them to miss classes

The majority of students expressed agreement with the following statements: (i) the course content was perceived as excessive in relation to the time available for study (568 responses (72.0%; 95% CI 0.693, 0.750)); (ii) the faculty members were perceived as lacking motivational strategies (414 responses (52.4%; 95% CI 0.492, 0.559)); (iii) a lack of coordination was perceived in courses taught by multiple professors (412 (52.2%; 95% CI 0.489, 0.557)); (iv) almost half of respondents felt that professors’ teaching methods were ineffective (49.0%; 95% CI 0.457, 0.525); (v) the faculty members are not interested in students’ learning (369 responses (46.7%; 95% CI 0.434, 0.503)); and (vi) a significant number of students felt that professors’ teaching was limited to dictating lecture notes (364 responses (46.1%; 95% CI 0.428, 0.496)) (Fig. 3).

**Fig. 4:**
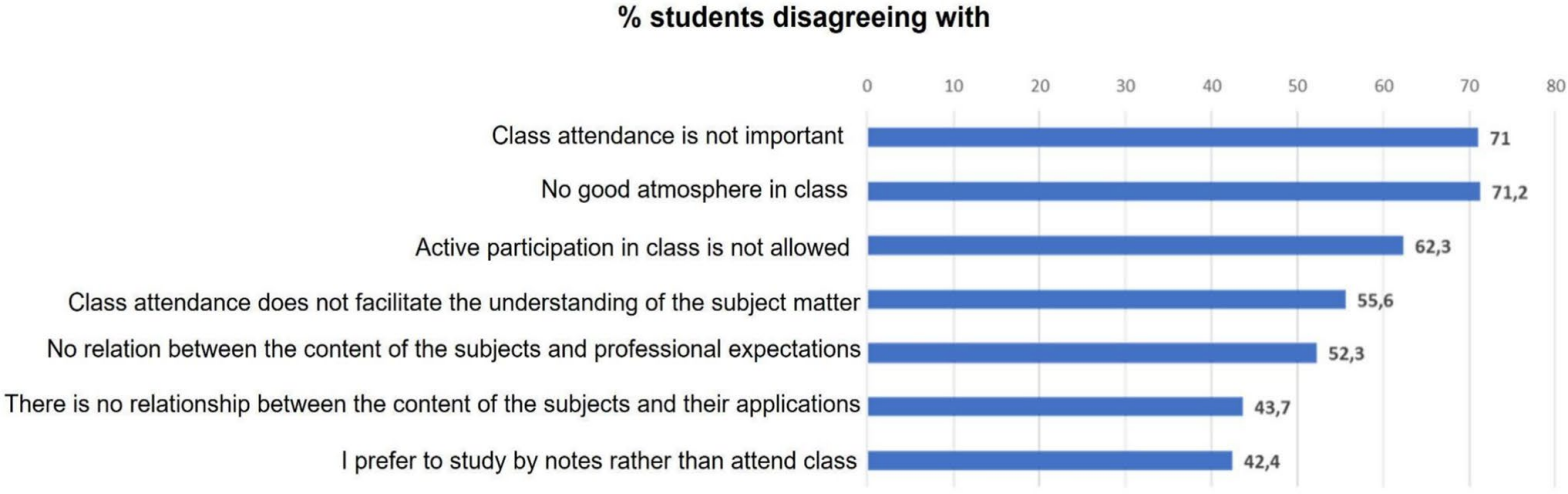
Reasons given by students on the aspects that motivate them to attend classes

Conversely, students largely disagreed with the following statements: (i) the importance of class attendance was strongly affirmed (457 responses (71.5%; 95% CI 0.686, 0.750)); (ii) a generally positive classroom environment was perceived (562 responses (71.2%; 95% CI 0.685, 0.743)); (iii) active participation in class was not perceived as restricted (492 responses (62.3%; 95% CI 0.593, 0.657)); (iv) class attendance was considered beneficial for subject comprehension (439 responses (55.6%; 95% CI 0.524, 0.591)); (v) course content was considered relevant to professional expectations (413 (52.3%; 95% CI 0.490, 0.558)); (vi) course content was not related to practical applications (345 responses (43.7%; 95% CI 0.402, 0.472)); and (vii) a preference for studying lecture notes rather than attending class was not widespread (335 (42.4%; 95% CI 0.391, 0.459)) (Fig. 4).

Regarding the open-ended responses, in which students were asked to list up to five reasons for not attending class in order of importance, we categorized the answers into the following groups:

1. Pedagogical deficiencies (reading directly from slides/notes, excessive and fast-paced information delivery, monotonous lectures, unengaging presentations, unclear explanations of the content, and an excessive number of instructors teaching certain courses).
2. Class materials are readily available (notes, slides) and, in some cases, the lectures themselves.
3. Lack of time to study the subjects.
4. Scheduling conflicts with practical sessions from other courses.
5. Discomfort associated with early morning classes.

Similarly, responses to the open-ended question asking students to list up to five reasons for attending class were grouped as follows:

1. Higher quality of classes (greater motivation from faculty, courses taught by a single professor, less monotonous classes, slower-paced explanations, and avoidance of reading directly from slides).
2. Greater interaction and student-faculty engagement.
3. Smaller class sizes.
4. More continuous learning through exercises, problem-solving, and exams.

Both students and teachers acknowledged the problem of absenteeism and agreed on the need to improve teaching methodology and increase classroom interaction. Similarly, they recognized that absenteeism is a significant problem. Professors mentioned that absenteeism is a serious issue, and students also indicated that attendance is important for understanding the subject matter. Both groups agreed that the teaching methodology needs improvement. Professors suggested innovating teaching methodologies and increasing classroom interaction, while students mentioned that professors limit themselves to dictating notes and do not have a proper methodology. The lack of interaction in class seems to be a problem for both groups. Professors proposed increasing interaction between professors and students, and students also indicated that classes should be more dynamic and participative. Both students and professors agreed that faculty motivation is crucial. Professors mentioned the need to motivate students, and students indicated that professors do not adequately motivate them.

However, there are differences in the perception of attendance and the reasons for absenteeism, as well as in suggestions for improving attendance. These differences highlight the importance of addressing the problem of absenteeism from multiple perspectives and involving both students and teachers in the search for effective solutions.

## Discussion

This study shows the significant absence of students from lectures and the numerous shortcomings they perceive in these lectures. The significant decrease in attendance observed throughout the semester highlights a concerning trend in medical education. Our data indicate that absenteeism is perceived as a significant issue by most faculty members. Faculty perspectives on medical student absenteeism have been primarily explored in a few key studies, not as much as students’ perceptions. Professors generally perceive student absenteeism as a significant problem and a challenge in curriculum delivery within education globally.

Our data extracted from the teacher survey results align significantly with the prevailing discourse in existing literature regarding teacher perceptions of student absenteeism. This convergence underscores the consistency and general patterns observed within medical education.

The interpretation of absenteeism in medical education is multifaceted, not reducible to a single cause. Instead, it appears to be a symptom of a complex interplay between institutional pedagogical shortcomings and a discernible shift in student values toward more self-directed learning. On one hand, our findings, consistent with prior literature [1], reveal that perceived deficiencies in teaching quality are a salient contributor to student absenteeism. The prevalence of non-interactive and monotonous teaching styles, coupled with student perceptions regarding excessive content, lack of faculty motivational strategies, poor coordination across courses, and ineffective teaching methods—such as merely dictating notes [12–14]—points directly to the need for pedagogical reform and a reevaluation of current teaching practices [12, 15]. Institutions should invest in training faculty with the tools and strategies necessary to foster active learning. Providing faculty with training in student-centered pedagogical approaches can enhance the quality of instruction and increase student engagement [16].

On the other hand, absenteeism can also be interpreted as a reflection of evolving student learning preferences. The availability of recorded lectures and extensive online resources has contributed to a shift in how students choose to learn. Many perceive that academic success is attainable even without regular in-person attendance, opting for self-directed and collaborative learning methodologies that they consider more effective or better suited to their lifestyles. This perspective, in line with Self-Determination Theory (SDT), suggests that when the learning environment does not support autonomy and competence, students may seek alternatives that provide them with greater control over their formative process, manifesting as reduced class attendance. This dichotomy in the causes of absenteeism leads us to explore the inherent tension between mandatory attendance policies and the promotion of student autonomy, a debate of considerable relevance in contemporary medical education.

While many medical institutions implement strict attendance policies [17–19], arguing their essentiality for the acquisition of knowledge and the development of non-cognitive skills such as professionalism, communication, and teamwork, rigid enforcement of attendance might be counterproductive if not accompanied by a clear value proposition from the lectures. From the perspective of Self-Determination Theory (SDT), a learning environment perceived as overly controlling or disengaging may undermine intrinsic motivation, leading to disaffection and, ultimately, absenteeism [20]. Students, in their pursuit of satisfying their needs for autonomy and competence, may devalue traditional lectures if they do not perceive significant added value that justifies in-person presence beyond mere content acquisition. Therefore, instead of coercion [21], there is a need to foster a culture of engagement and continuous assessment that encourages active participation, peer-to-peer and faculty-student interaction, and the application of knowledge in real contexts. Restructuring class formats to incorporate interactive elements and practical applications could be key to reconciling the need for presence with student autonomy, making attendance a choice driven by perceived value rather than a mere obligation.

The development of non-cognitive skills, such as professionalism, empathy, and the ability to work in teams, is fundamental for effective and patient-centered healthcare practice. The arguments for and against mandatory attendance often overlook the value gained from being in the classroom itself, focusing primarily on academic performance. Therefore, class attendance and active participation in classroom activities can significantly contribute to the comprehensive training of future medical professionals, beyond the mere acquisition of theoretical knowledge [12].

The analysis of student perspectives reveals a complex interplay of factors contributing to absenteeism within medical education. The perception that academic success is attainable independent of regular attendance, coupled with the accessibility of digital resources, raises pertinent questions regarding the perceived value of in-person instruction [12, 15, 16]. Although we did not conduct statistical tests to compare attendance across academic years, the descriptive data collected during the two assessment points revealed notable differences. Specifically, first-year students exhibited the highest attendance rates, while fifth-year students showed the lowest. These patterns suggest a potential trend of decreasing attendance as students advance through the program, which may reflect evolving attitudes toward lecture-based learning or increased reliance on alternative study methods. Academic factors emerge as significant contributors to student disengagement. The monotony of lectures, perceived deficiencies in teaching quality, and inconvenient scheduling are consistently cited as substantial barriers. Furthermore, personal challenges, including fatigue, examination-related stress, and health concerns, are compounded by logistical obstacles such as transportation issues and inadequate study facilities. Student recommendations underscore a desire for enhanced interactivity, flexibility, and communication. The preference for smaller group sessions and dynamic learning methodologies highlights the need for a paradigm shift in educational delivery. The expressed need for improved faculty communication emphasizes the importance of cultivating supportive and robust student-faculty relationships. Collectively, these findings underscore the necessity for medical education institutions to reassess their teaching strategies and student support systems. It is imperative to cultivate a learning environment that is engaging, flexible, and responsive to student needs. This entails not only the adoption of innovative methodologies but also a concerted effort to address the personal and logistical factors that impact student attendance and well-being.

A significant limitation of this study lies in the fact that most of the opinions collected are from students who were present in class on the day attendance was recorded and the survey was distributed. Consequently, it is highly probable that we missed valuable insights from those students who rarely attend class, who are arguably the most interesting and relevant group in the context of chronic absenteeism. This potential underrepresentation is inferred by observing that, while 71.5% of responding students reported attending class more than 80% of the time, only 16.2% believed their peers attended more than 80% of the time. This discrepancy underscores the possible gap between self-reported attendance and the widespread perception of the problem.

Although conscious efforts were made to mitigate this bias, such as circulating the survey through the student delegation with the aim of capturing impressions from those not present in the classroom on the day of the measurement, the possibility remains that habitually absent students were not reached, and thus, their experiences and reasons for non-attendance remain underrepresented in the findings. The impact of this bias could mean that the causes of absenteeism identified in this study are more representative of students with moderate absenteeism, while the underlying factors for severe or chronic absenteeism might differ substantially (for example, related to more complex personal issues or deeper academic disengagement). Future research should consider specific methodological strategies, such as focused qualitative interviews or more intensive longitudinal tracking, to capture the perspectives of students who frequently miss class, which is critical for obtaining a comprehensive and representative understanding of the issue.

## Conclusions and Recommendations

Student absenteeism at the Faculty of Medicine of the University of Granada represents a significant challenge that requires a comprehensive, evidence-based approach.

The findings of this study provide a comprehensive understanding of medical student absenteeism, highlighting its multifaceted nature from both student and faculty perspectives. Our research indicates that absenteeism is a complex issue driven by a combination of pedagogical shortcomings, evolving student learning preferences, and personal and logistical challenges. The observed decrease in attendance as students’ progress through their academic years, coupled with their preference for self-directed learning and the availability of digital resources, underscores a critical juncture in medical education.

Based on these insights, we propose three key actionable recommendations for medical education institutions, emphasizing their feasibility for immediate implementation:

- Enhance Pedagogical Innovation and Interactivity: It is crucial to redesign traditional lecture formats to incorporate more interactive and student-centered methodologies. This includes integrating strategies such as case-based discussions, flipped classrooms, and problem-based learning. Feasibly, this can be achieved through targeted faculty development programs that equip educators with the skills and resources to implement these engaging approaches, making in-person attendance more valuable and less perceived as passive information transfer.
- Foster a Supportive and Engaging Learning Environment: Institutions should prioritize initiatives that improve faculty-student communication and interaction, creating a collaborative atmosphere. This involves promoting accessible office hours, mentorship opportunities, and utilizing communication technologies to facilitate engagement. The feasibility lies in encouraging a culture where faculty are supported in dedicating time to student interaction and where institutional platforms promote easy communication.
- Re-evaluate Attendance Policies with Autonomy in Mind: While acknowledging the importance of attendance for developing non-cognitive skills crucial in medicine, policies should be reconsidered to balance mandatory presence with student autonomy. A feasible approach involves moving towards a system that emphasizes the value proposition of attending lectures through enhanced quality and interactivity, rather than relying solely on coercive measures. This encourages attendance based on perceived benefit, fostering intrinsic motivation rather than mere compliance.

These findings, derived from the context of the University of Granada, are highly transferable to other medical education institutions, particularly within Spain and across Europe, given common pedagogical structures and student demographics. The challenges and student preferences identified here resonate broadly with the ongoing shifts in higher education.

Ultimately, addressing medical student absenteeism effectively requires institution-wide interventions and policy changes that are firmly rooted in evidence. This study provides a robust foundation for such strategic re-evaluations, urging medical faculties to adapt their educational models to foster greater student engagement, promote active learning, and cultivate a more responsive and supportive academic environment for future healthcare professionals.

## Supplementary Information

Below is the link to the electronic supplementary material.

ESM1 (PDF 180 KB)

## Data Availability

All data generated or analyzed during this study are included in this published article and its supplementary information files.
