# Supplementary material for: Exploring Absenteeism in Medical Education: Perceptions of Students and Professors at the University of Granada
Source: Med Sci Educ. 2025 Sep 20;35(6):2861–9. doi: 10.1007/s40670-025-02482-w (PMC12960864; doi:10.1007/s40670-025-02482-w)
Supplement: Supplementary file 1 — (PDF 180 KB) [file 40670_2025_2482_MOESM1_ESM.pdf]

TEACHER SURVEY

Participant Information:

Years of teaching experience
Gender
Age
Subjects
Course
- First Year
- Second Year
- Third Year
- Fourth Year
- Fifth Year

Questions:

Student absenteeism in this faculty is:
A serious problem.
A relative problem.
Not a problem at all.
Other

Student absenteeism in this faculty:
Occurs in all faculties and has little solution.
Could be solved by taking daily attendance.
Would be solved if the teaching staff were of better quality.
None of the above.

Which of these solutions do you consider most important to reduce student absenteeism in this faculty?
Smaller groups of students.
Classes with greater interaction.
Students teaching some of the classes.
None of the above.

Do you wish to make any additional comments?

STUDENT SURVEY
Data

- Male ❑ Female ❑ Age ❑
- Grade point average up to the current course:
- Pass ❑
- Good ❑
- Outstanding ❑
- Number of subjects you are enrolled in:
- First year ❑ - Second year ❑
- Third year ❑ - Fourth year ❑
- Fifth year ❑ - Electives ❑
- Free configuration ❑
- I travel from another town to attend class: Yes ❑ No ❑
- Usual place of residence during the course:
- Family home ❑
- Other ❑
- I enrolled in the degree as: 1st choice ❑ 2nd choice ❑ 3rd choice ❑
- In general, and considering the subjects in which I am enrolled, I attend class:

Always ❑ Frequently ❑ Sometimes ❑ Never ❑

WRITE IN ORDER OF IMPORTANCE UP TO FIVE REASONS THAT INFLUENCE YOU NOT TO ATTEND SOME OR SEVERAL CLASSES
1.- ....
2.- ....
3.- ....
4.- ....
5.- ....

WRITE IN ORDER OF IMPORTANCE UP TO FIVE REASONS THAT YOU THINK WOULD BE NECESSARY FOR YOU TO ATTEND CLASS REGULARLY
1.- ....
2.- ....
3.- ....
4.- ....
5.- ....

I DO NOT ATTEND CLASS WHEN OR BECAUSE

1. Attendance is not important.

Strongly agree ❑ Agree ❑ Disagree ❑ Strongly disagree ❑

2. Attendance does not facilitate my understanding of the subject.

Strongly agree ❑ Agree ❑ Disagree ❑ Strongly disagree ❑

3. I prefer to study from notes than to attend class.

Strongly agree ❑ Agree ❑ Disagree ❑ Strongly disagree ❑

4. Keeping up with the class pace implies too much effort.

Strongly agree ❑ Agree ❑ Disagree ❑ Strongly disagree ❑

5. Attendance is not taken.

Strongly agree ❑ Agree ❑ Disagree ❑ Strongly disagree ❑

6. Close to exams, I prefer to dedicate time to studying.

Strongly agree ❑ Agree ❑ Disagree ❑ Strongly disagree ❑

7. The volume of subject content does not correspond to the limited time available to study it.

Strongly agree ❑ Agree ❑ Disagree ❑ Strongly disagree ❑

8. Class attendance does not help me to pass.
Strongly agree ❑ Agree ❑ Disagree ❑ Strongly disagree ❑
9. The subject content is very difficult.
Strongly agree ❑ Agree ❑ Disagree ❑ Strongly disagree ❑
10. The subject content is very easy.
Strongly agree ❑ Agree ❑ Disagree ❑ Strongly disagree ❑
11. I prefer to attend an academy or private lessons.
Strongly agree ❑ Agree ❑ Disagree ❑ Strongly disagree ❑
12. I am not allowed to participate actively in class.
Strongly agree ❑ Agree ❑ Disagree ❑ Strongly disagree ❑
13. I am worried about being asked questions in class.
Strongly agree ❑ Agree ❑ Disagree ❑ Strongly disagree ❑
14. The teaching staff is limited to dictating notes.
Strongly agree ❑ Agree ❑ Disagree ❑ Strongly disagree ❑
15. The teaching staff does not have a good methodology.
Strongly agree ❑ Agree ❑ Disagree ❑ Strongly disagree ❑
16. The relationship between the subject content and its possible applications is not conveyed to me in class.
Strongly agree ❑ Agree ❑ Disagree ❑ Strongly disagree ❑
17. The materials and resources used in the classroom are inadequate.
Strongly agree ❑ Agree ❑ Disagree ❑ Strongly disagree ❑
18. In class, I am not helped to learn, only information is transmitted to me.
Strongly agree ❑ Agree ❑ Disagree ❑ Strongly disagree ❑
19. The teaching staff is not interested in my learning process.
Strongly agree ❑ Agree ❑ Disagree ❑ Strongly disagree ❑
20. The explanations are not adapted to my level of knowledge and understanding.
Strongly agree ❑ Agree ❑ Disagree ❑ Strongly disagree ❑
21. My failures are emphasized and my successes are minimized in class.
Strongly agree ❑ Agree ❑ Disagree ❑ Strongly disagree ❑
22. The subject is taught by more than one teacher and there is a lack of coordination.
Strongly agree ❑ Agree ❑ Disagree ❑ Strongly disagree ❑
23. The evaluation is arbitrary and subjective.
Strongly agree ❑ Agree ❑ Disagree ❑ Strongly disagree ❑
24. The evaluation does not correspond to what was explained in class.
Strongly agree ❑ Agree ❑ Disagree ❑ Strongly disagree ❑
25. The student's effort in the classroom is not valued.
Strongly agree ❑ Agree ❑ Disagree ❑ Strongly disagree ❑
26. The teaching staff does not motivate.
Strongly agree ❑ Agree ❑ Disagree ❑ Strongly disagree ❑
27. Occasionally, a student is criticized or singled out.
Strongly agree ❑ Agree ❑ Disagree ❑ Strongly disagree ❑
28. There is not a good atmosphere in the class group.
Strongly agree ❑ Agree ❑ Disagree ❑ Strongly disagree ❑
29. The teaching staff is not interested in getting to know the students.
Strongly agree ❑ Agree ❑ Disagree ❑ Strongly disagree ❑
30. I do not find a direct relationship between the subject content and my training expectations.
Strongly agree ❑ Agree ❑ Disagree ❑ Strongly disagree
31. Schedules influence my non-attendance in class. (Check the options that match your opinion)

- Because they overlap ❑
- Because there are free hours between classes ❑
- Because they are in the afternoon ❑
- Because they are morning and afternoon ❑
- Because there are many classes in a row ❑
- Because I have to travel from another town ❑
- Because I combine my academic activity with professional obligations ❑
- Because I combine my academic activity with family obligations ❑
- In case of "abandoning" the subject, I do it:
- At the beginning ❑
- Halfway through the course or semester ❑
- In case of never attending class, I still intend to take the final exam: Yes ❑ No ❑
